# Correlated activity of cortical neurons survives extensive removal of feedforward sensory input

**DOI:** 10.1038/srep34886

**Published:** 2016-10-10

**Authors:** Katharine A. Shapcott, Joscha T. Schmiedt, Richard C. Saunders, Alexander Maier, David A. Leopold, Michael C. Schmid

**Affiliations:** 1 Ernst Strüngmann Institute (ESI) for Neuroscience in cooperation with Max Planck Society, 60528 Frankfurt, Germany; 2 Laboratory of Neuropsychology, National Institute of Mental Health, Bethesda, Maryland 20892, USA; 3 Vanderbilt University, Department of Psychology, Nashville, Tennessee 37240, USA; 4 Neurophysiology Imaging Facility, National Institute of Mental Health, National Institute of Neurological Disorders and Stroke, and National Eye Institute, Bethesda, Maryland 20892, USA; 5 Institute of Neuroscience, Newcastle University, Framlington Place, Newcastle upon Tyne, NE2 4HH, UK

## Abstract

A fundamental property of brain function is that the spiking activity of cortical neurons is variable and that some of this variability is correlated between neurons. Correlated activity not due to the stimulus arises from shared input but the neuronal circuit mechanisms that result in these noise correlations are not fully understood. Here we tested in the visual system if correlated variability in mid-level area V4 of visual cortex is altered following extensive lesions of primary visual cortex (V1). To this end we recorded longitudinally the neuronal correlations in area V4 of two behaving macaque monkeys before and after a V1 lesion while the monkeys fixated a grey screen. We found that the correlations of neuronal activity survived the lesions in both monkeys. In one monkey, the correlation of multi-unit spiking signals was strongly increased in the first week post-lesion, while in the second monkey, correlated activity was slightly increased, but not greater than some week-by-week fluctuations observed. The typical drop-off of inter-neuronal correlations with cortical distance was preserved after the lesion. Therefore, as V4 noise correlations remain without feedforward input from V1, these results suggest instead that local and/or feedback input seem to be necessary for correlated activity.

Repeated presentations of the same stimulus to cortical neurons results in highly variable responses^1^,^2^. Some of this variability is shared among neurons^3^, resulting in correlated variability which likely influences how well information can be read-out by downstream target neurons receiving the correlated input^4^,^5^,^6^. Despite the theoretical importance of these “noise” correlations their origin is not currently known. Correlated neuronal variability could arise from multiple possible brain circuit mechanisms, including common feedforward projections from lower areas, feedback projections from higher areas and the local connectivity within an area. Previous findings have lent indirect support for either one of these scenarios^1^,^2^,^6^,^7^,^8^,^9^,^10^,^11^,^12^,^13^,^14^,^15^. Thus, shared neural variability appears to derive from multiple, parallel mechanisms that are not yet fully understood. A classic view of correlated activity emphasizes the potential role of shared feedforward input^1^,^14^,^16^.

In the present study we investigated the effect of surgically removing the primary sensory feedforward input, arising from area V1, on correlated firing of neurons in the primate visual cortical area V4. This experimental manipulation examines whether correlated firing within a given cortical area is inherited from earlier stages of the hierarchy, as V4 receives direct as well as indirect feedforward input from V1^17^,^18^,^19^. We have previously shown, using both functional magnetic resonance imaging as well as in neurophysiological recordings, that neuronal activity in areas V2 and V4 is reduced by ~80% on average without V1 input, but nevertheless remains significantly responsive to visual stimuli^20^,^21^,^22^. For the current analysis in V4, we hypothesized that if correlated firing were the result of a cascade of shared feedforward input alone, then removing the V1 input would be expected to decrease V4 noise correlations. To reduce the influence of stimulus induced effects, we sampled spontaneously occurring noise correlations in monkeys fixating a gray computer screen.

## Results

### Trial-by-trial neuronal correlations

**Figure 1:**
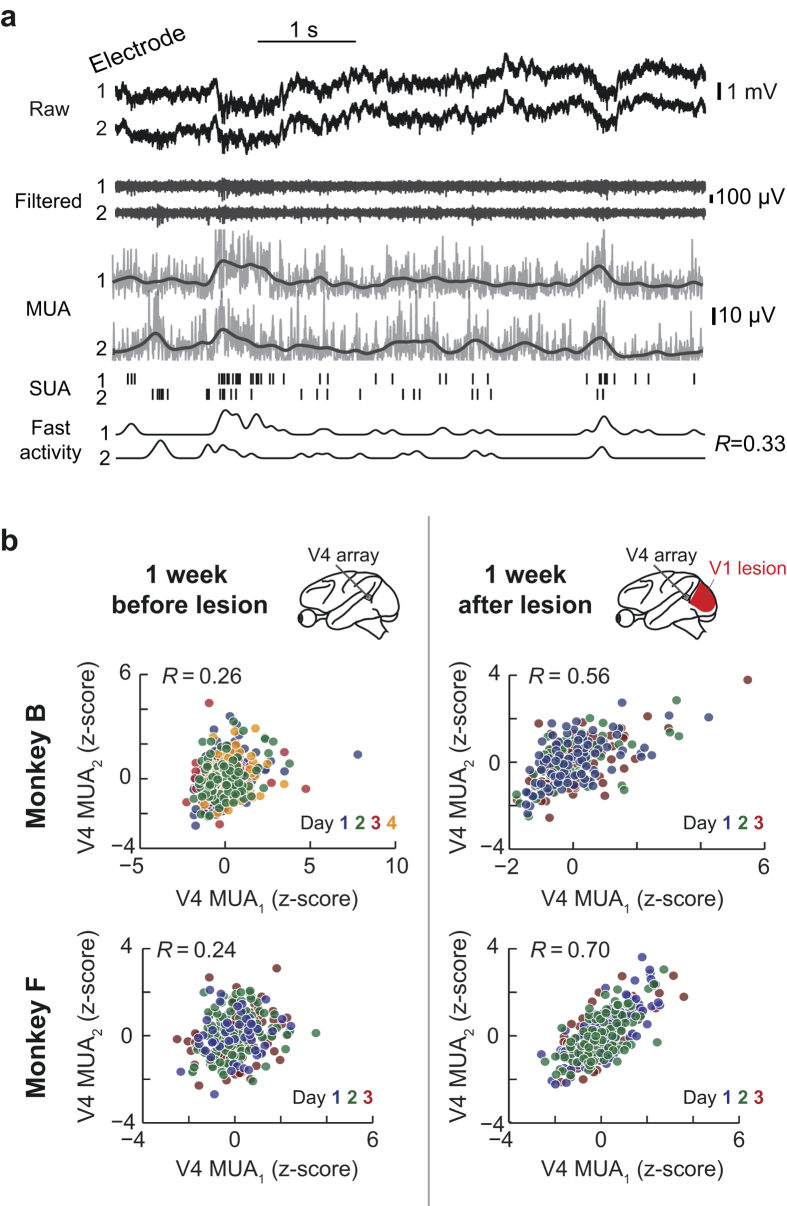
Correlated firing variability pre and post V1 lesion. (**a**) Multi-unit-activity (MUA) and spike train correlation calculation. Shown here are two simultaneously recorded electrode channels. Note that all signals show on-going variability, of which some is correlated between the two channels. Steps to calculate these signals from the raw data were as follows: To create MUA the raw data was band-pass filtered between 300–12000 Hz (“Filtered” in plot). This was then rectified and downsampled to create the MUA (gray line is the MUA, black line is the MUA smoothed). Short vertical lines are spikes extracted using a threshold (SUA). Note that the change in rate of these spikes closely follows the MUA. Spike train correlation was calculated by taking the convolution with a Gaussian window (standard deviation of 25 ms) of the spike train of two neurons (“Fast Activity”) and calculating the correlation coefficient^5^. In this example trial the fast correlation of the SUA was 0.33. (**b**) Example rate correlation calculation. Shown is the z-scored MUA for channel pairs from monkey B and monkey F and the calculated Pearson’s correlation coefficients. Colors indicate different recording sessions, which relative to the lesion (day 0) are for monkey B before lesion −6, −3, −2 and −1 and after lesion 4, 5 and 6 and for monkey F before lesion −4, −2 and −1 and after lesion 2, 4 and 6. Note that while pre and post lesion sessions overlay, between pre and post there is an increase in the correlation coefficient in these representative examples.

**Figure 2:**
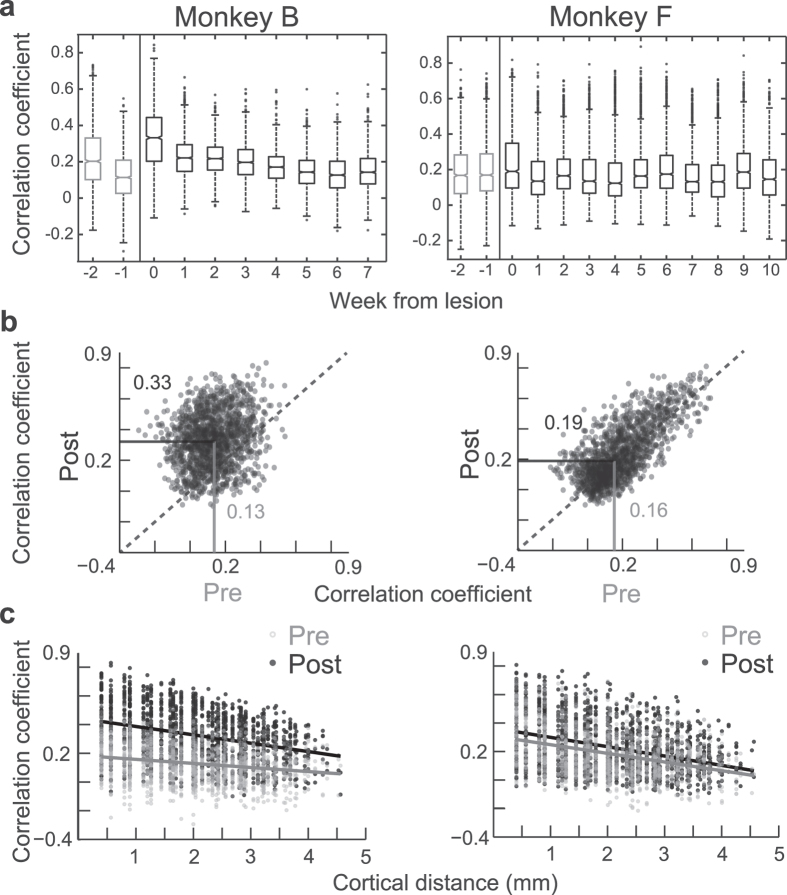
V1 lesion causes an increase in trial-by-trial V4 rate correlation. (**a**) Correlated firing over weeks. The correlation was calculated for each pair for each day and averaged over each week of the recordings. Boxplots show the distribution of all pairs each week. Week 0 is the week of the lesion. (**b**) Scatter plots of the rate correlation calculated for each channel pair 1 week pre and 1 week post V1 lesion (week −1 and week 0 in panel a). In both monkeys a highly significant shift upwards is evident. Median values are printed and indicated by the lines. (**c**) Rate correlation with channel distance for pre and post lesion. Two separate linear fits for data 1 week pre and 1 week post V1 lesion are plotted over the data points. Note that the slope is negative both pre and post lesion in both monkeys. See also Supplementary Figs S1–S3.

**Figure 3:**
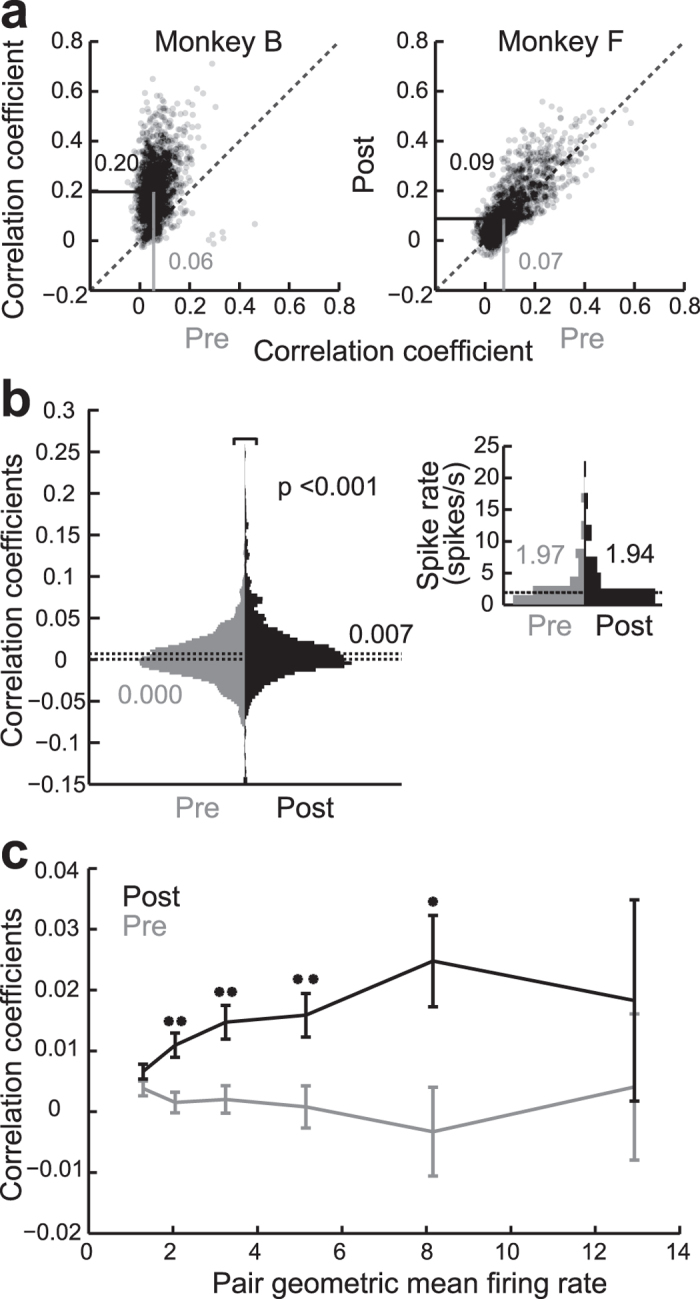
V1 lesion causes an increase in MUA and SUA time course correlations. (**a**) Scatter plots of the short timescale MUA rate correlation calculated for each channel pair pre and post V1 lesion. In both monkeys a highly significant shift upwards is evident. Median values are printed and indicated by the line. (**b**) Histogram of SUA correlation coefficients pre and post lesion. Histogram is the kernel density estimation. Inset is spike rate distribution pre and post lesion. Median values are printed and indicated by the line. (**c**) Average correlation coefficients for SU neuron pairs binned by the pair geometric mean firing rate. Error bars are SEM. Note that the spike train correlation from pre to post is increased in all bins. Stars show significance levels, **p < 0.01, *p < 0.05. For each bin the Wilcoxon rank sum one-sided test was run, p values respectively were; *p* = 0.079, *p* < 0.001, *p* = 0.002, *p* = 0.005, *p* = 0.025, *p* = 0.439; Ns respectively were*; n*(pre, post) = (252, 192), *n* = (276, 164), *n* = (214, 160), *n* = (115, 141), *n* = (34, 79), *n* = (6, 29).

Our first goal was to test how much neuronal activity was correlated between V4 neurons on a trial-by-trial basis, that is, on correlations that occur over multiple seconds or longer and that are likely influenced by arousal and attention^3^,^23^. To test this experimentally, neurophysiological recordings were first made under intact V1 conditions, from electrode arrays chronically implanted in area V4. A surgical aspiration lesion was then subsequently performed in area V1 allowing measurement of V4 activity without V1 input. We analyzed data that were acquired while the monkeys visually fixated for 800 ms a small dot of light on a grey background of a computer monitor. To estimate interneuronal correlations, the average multi-unit activity (MUA) rate for each trial^24^ (see Methods) was calculated for each electrode (Fig. 1). The correlation of these pooled values across trials was calculated for each electrode pair (*n* = 1596 pairs in monkey B, *n* = 1596 pairs in monkey F) to give two MUA rate correlation numbers per pair for each experimental session. This trial-by-trial MUA rate estimate within a session was pooled across each week before and after lesioning V1 until a maximum of 11 weeks (Fig. 2a). One week prior to the lesion procedure (*n* = 4 sessions in monkey B, *n* = 3 sessions in monkey F), the median of the electrode pairs’ noise correlations across sessions was similar in both monkeys (pre lesion median ± IQR = 0.126 ± 0.172 for monkey B and 0.157 ± 0.205 for monkey F, Fig. 2a). After the lesion, correlations appeared overall quite similar across time points and monkeys compared to baseline conditions with V1 intact. Notably however, within the first week after the V1 lesion (*n* = 3 sessions in monkey B, *n* = 3 sessions in monkey F), correlations were increased compared to the week immediately pre lesion in both monkeys. This was particularly strong in monkey B (post lesion median ± IQR = 0.327 ± 0.242), but was also present to a smaller extent in monkey F (0.186 ± 0.251) (Fig. 2b; Wilcoxon rank sum test; *p* < 0.001 and *p* < 0.001, respectively). However, in monkey F other week-to-week increases post-lesion were larger than the increase from pre to post lesion. In everything that follows, we focus our further analysis on this increased correlated variability during the first week after lesioning V1 compared to the week pre lesion. In a first step, we checked that increased noise correlations were not due to a lesion-induced systematic change in fixational eye movements (see Supplementary Fig. S1). We additionally still observed increased trial-by-trial correlation post lesion (see Supplementary Fig. S2) after removing the effects of long term correlations over multiple trials. Further, correlations also increased from pre to post lesion for spike count correlations between single-unit pairs assessed in monkey B (see Supplementary Fig. S3). The magnitude of these single-unit correlations is far weaker than those of the multi-units, however, it is of similar magnitude to previously reported values for single-units with very low firing rates (Fig. 3b insert)^5^,^16^. In line with the MUA finding, single-unit correlations significantly increased from pre to post lesion. From this analysis we can therefore conclude that correlated neuronal activity in V4 was not only present in the absence of feedforward input from V1, but that it displayed a surprising increase in at least one monkey compared to conditions with intact V1 input during the first week after lesioning V1.

### Neuronal correlations as a function of cortical distance

It is known that neurons share more common feedforward input with their immediate neighbours than with neurons that are more distal^12^,^13^,^25^,^26^. This is thought to contribute to the falloff in correlated activity as a function of cortical distance^12^,^25^. We therefore wondered whether this cortical distance function of correlated activity would change in V4 following the V1 lesion. To this end, we first plotted the pre lesion MUA rate correlation values against the distance between electrodes. This analysis confirmed the expected falloff of correlated activity with distance during V1 intact conditions: A linear regression fit to the pre lesion data showed a significant inverse relationship between correlation and interelectrode distance with a slope of −0.028 ± 0.003 μm^−1^ in monkey B and a slope of −0.061 ± 0.004 μm^−1^ in monkey F (F-test(1, 1594); *p* < 0.001 and *p* < 0.001, respectively). Interestingly however, the resulting distance function persisted after the lesion, indicating that this feature of noise correlations could not derive either directly or indirectly from V1 feedforward input (Fig. 2c): After the lesion, not only was a similar falloff present, but the slope became steeper reaching −0.058 ± 0.003 μm^−1^ in monkey B and −0.066 ± 0.004 μm^−1^ in monkey F (F-test(1, 1594); *p* < 0.001 and *p* = 0.001, respectively). The slope steepening following the lesion was significant for monkey B (ANCOVA interaction (1, 3188); standard error = 0.003; p < 0.001), but not for monkey F (ANCOVA interaction (1, 3188); standard error = 0.003; p = 0.403). The finding that trial-by-trial correlations persist in the absence of feedforward input and that they maintain their spatial specificity is in line with the view that such correlations are induced by fluctuations in attention or arousal^3^,^23^.

### Within trial neuronal co-fluctuation

The results presented so far revealed how spiking averaged over an 800 ms time-window was correlated between electrodes on a trial-by-trial basis before and after removing feedforward input from V1. These correlations therefore reflect influences that occur over the course of seconds or slower and are likely influenced by behavioral or brain state. But correlations in neuronal activity also arise much faster, on the millisecond time-range within a behavioral trial, likely reflecting the local processing of cortical neurons. We therefore extended our analysis on correlated activity to the fast activity changes that occurred within the 800 ms fixation period. To this end we measured the average MUA during 50 ms wide time windows and assessed the extent to which MUA spiking changes were correlated between electrode pairs within the trial until the end of the 800 ms long fixation period. We found again that prior to the lesion, the median of the electrode pairs’ short timescale correlations were similar in both monkeys (median ± IQR = 0.058 ± 0.057 for monkey B and 0.074 ± 0.112 for monkey F, Fig. 3a). After the V1 lesion, as found in the other analysis, there was a significant *increase* in within trial rate correlations among different electrode sites in V4, which was again particularly strong in monkey B (0.1971 ± 0.151), but was also present in monkey F (0.089 ± 0.151) (Fig. 3a; Wilcoxon rank sum test; *p* < 0.001 and *p* < 0.001, respectively).

To obtain a more direct measure of correlated within-trial neuronal activity, we additionally performed spike train correlations on 92 single units (897 simultaneously recorded pairs) pre lesion (days −8, −2 and −1 relative to lesion) and 83 single units (768 simultaneously recorded pairs) post lesion (days 4, 5 and 8 relative to lesion) in monkey B. This analysis was again done on the data recorded while the monkey fixated a small point on an otherwise gray screen. We assessed the correlation of single unit activity (SUA) convolved with a 25 ms standard deviation Gaussian and averaged over trials^5^. Only well isolated single units were used for the analysis. Correlations were again found to be significantly increased post lesion. Spike train correlations increased from 0.001 ± 0.031 pre lesion to 0.007 ± 0.033 post lesion (Fig. 3b; Wilcoxon rank sum; *p* < 0.001). When neuron pairs were binned by their geometric mean firing rate (Fig. 3c) correlated spiking from pre to post lesion was significantly increased in all but the first and last bins (Fig. 3c). Importantly, the overall single unit spike rates of neurons used to calculate the spike train correlations (see Methods) were not significantly different from pre (1.87 ± 2.86 spikes/s) to post (1.97 ± 5.47 spikes/s) lesion (Wilcoxon rank sum; *p* = 0.342). Similarly, the variability for all neurons did not change from pre to post lesion when measured by the Fano factor or the squared coefficient of variation of the ISIs (CV^2^). The Fano factor was median ± IQR = 1.85 ± 0.851 pre lesion and 1.97 ± 0.850 post lesion (Wilcoxon rank sum; *p* = 0.174), while the CV^2^ was 0.715 ± 0.473 pre lesion and 0.764 ± 0.332 post lesion (Wilcoxon rank sum; p = 0.1355). However, as there were only 14 of 92 single unit neurons pre lesion and 22 of 83 single unit neurons post lesion with an average firing rate greater than 5 spikes/s (as recommended in Nawrot *et al.*^27^); this may therefore not be a reliable measure of the true variability within this dataset. In conclusion, our results on the correlated variability that occurs at short timescales extend and confirm our observations on the trial-by-trial based analysis, namely that correlated neuronal activity on both time scales persists in V4 despite the absence of V1 feedforward input.

## Discussion

In this study we have shown that a lesion to the primary visual cortex, through which almost all visual information enters cortex, and which projects directly and indirectly to V4 among other areas^28^, leads to an increase in correlated noise in V4, in one monkey and no change or a small increase in another. The increase was found in MUA at both long and short timescales and in SUA for different firing rate ranges, and without an increase in the overall spike rate magnitude and Fano factor pre to post lesion, i.e., effects that could cause correlation increase^12^,^22^,^29^. This result is surprising, as correlated neuronal firing is often at least in part thought to result from a cascade of common sensory feedforward input^1^,^14^,^16^ and V4 correlated activity would therefore be predicted to decrease after a V1 lesion. Our results however, argue against such a scenario. Thus, noise correlations in higher-level cortex, at least in V4, cannot be explained on the basis of feedforward sensory input from V1 alone. What might be the sources of correlated activity if it remains without feedforward mechanisms and why did we observe a correlation increase in at least one monkey following the removal of V1 input? Previously, Ecker and colleagues^16^ suggested that elevated firing correlations may result from slow trial-by trial drifts of neuronal excitability due to varying levels of alertness or arousal. However, such slow modulations are unlikely to explain our results alone, as we have found the increase simultaneously on timescales of less than 800 ms and more than 5 seconds. Other influences on correlated firing might instead comprise aspects of the local microcircuit or secondary input from other cortical as well as subcortical structures. Changes in the local microcircuit, in the context of our experiments, may be due to intrinsic plasticity caused by the altered input situation^30^,^31^ or due to selective strengthening of certain components, for example local horizontal connections^32^, which have been suggested to cause short timescale neuronal correlation within V4^12^,^13^. Alternatively, feedback from higher cortical areas in particular has been suggested to be an important factor in controlling correlation levels based on findings that noise correlations in V4 depend on the extent of top-down cognitive engagement during attention and learning tasks^6^,^10^,^11^,^15^.

In the context of the current experiments it is conceivable that top-down or feedback influences may have contributed to our results in several ways. At the behavioral level, it is important to keep in mind that the strongest increase in monkey B was observed directly after the lesion, as was the small increase in monkey F. It is known that monkeys during this period are blind and cannot saccade to visual stimuli presented to the scotoma^22^,^33^,^34^. In line with the observation that noise correlations depends on the level of top-down engagement^6^,^10^, the increase in correlated activity observed in the present study might therefore reflect the altered perceptual status of the monkeys immediately following the V1 lesion. At the neural level, oscillations in the beta range have been recently reported as markers for cortical feedback signaling^35^,^36^. We have previously reported prominent alpha/beta oscillations (12–20 Hz) in V4 following a V1 lesion^37^, perhaps indicating unmasked or upregulated feedback influences that could possibly contribute to the increase in V4 correlation levels we observed in the present analysis. While ultimately several scenarios shaping correlations seem possible at this point, we have shown that even with reduced feedforward sensory input, noise correlations remain substantial in extrastriate visual cortex.

## Methods

Methods as in Schmid *et al.*^22^ and described briefly below.

### Subjects

Two healthy female *Macaca mulatta* with prior V1 lesions in the right hemisphere were used in the study. All procedures were in accordance with the Institute for Laboratory Animal Research Guide for the Care and Use of Laboratory Animals and approved by the Animal Care and Use Committee of the National Institute of Mental Health.

### Surgical procedures

The procedures used were as described in Schmid *et al.*^22^. A chronic 10 × 10 “Utah” array of microelectrodes (Blackrock Microsystems) was inserted into visual area V4. In a subsequent surgery, following the first period of recordings, an aspiration lesion was performed in V1 covering the central 7 degrees of the visual field. The extent of the lesion was confirmed in histology carried out post mortem.

### Array implantation

The left hemisphere that was studied here was intact at the beginning of the study. A large occipital bone flap over the left hemisphere was created to warrant access to areas V1 and V4. The “Utah” array was subdurally inserted on the prelunate gyrus, ~2 mm dorsal of the lateral tip of the inferior occipital sulcus, near the entrance of the superior temporal sulcus. Each microelectrode was 1.5 mm long with a tip radius ranging from 3 to 5 μm, and an interelectrode spacing of 400 μm. After array implantation, dura and bone flap were sutured back in place and covered with the skin. Electrical reference was a wire located subdurally over the parietal cortex.

### Behaviour

Monkeys completed a passive viewing task of which 800 ms was a baseline fixation period without visual stimulation, which was used for analysis. They had to maintain fixation within a 0.75–1° radius of the fixation point, and after 3000 ms of fixation a variable amount of juice reward was delivered. Typically ~80 of such trials were performed per session. Eye movements were monitored either using the scleral search coil technique or via infrared-based tracking of the pupil. Stimuli were presented with a screen refresh rate of 60 Hz on a single LCD Samsung monitor (height 40 cm, width 65 cm) positioned at a viewing distance of 100 cm.

### Neurophysiological recordings

We recorded from a random selection of 64 channels of the 96 channels available from the array. Electrode impedances ranged between 150 and 1 MΩ at 1 kHz. Extracellular voltages were amplified, filtered between 0.1 Hz and 12 kHz, and digitized at 24, 414.1 Hz using a 64-channel RZ2 recording system (Tucker Davis Technologies).

### Estimating MUA activity

To obtain an estimate of multi-unit activity (MUA) (see Fig. 1)^24^, the raw signal was bandpass-filtered between 300 Hz and 12 kHz with a fourth order zero-phase Butterworth filter, rectified, low-pass filtered at 120 Hz with a sixth order zero-phase Butterworth filter, and downsampled to 256 Hz, yielding a quasi-continuous measure of high-frequency field power.

### Data analysis

All data were analyzed with the MATLAB (R2011a, MathWorks) toolbox FieldTrip^38^ and custom-written analysis scripts. The significance of differences between pre and post lesion was assessed using a Wilcoxon rank sum test for all correlation analyses. Values reported in the text are median ± interquartile range (IQR) for population measures and slope ± standard error for linear regression if not stated otherwise in the text. The histograms were calculated using kernel density estimation with a normal kernel. The bandwidth was chosen to be half of the optimal bandwidth for normal data to avoid over smoothing. To calculate the Fano Factor we took the spike count for each neuron on each trial and calculated the variance of these spike count per neuron and then divided it by the mean per neuron. To calculate the coefficient of variation squared (CV^2^) of the interspike intervals (ISI) we took the variance of the ISIs and divided it by the mean squared of the ISIs for each neuron on each trial. We then took the mean CV^2^ across all trials to get one CV^2^ value for each neuron.

### Single unit isolation

Unit activity was extracted by first high-pass filtering the signal with a median filter (0.5 ms window half-length) and then using a threshold of 1.3 SD. Units were isolated semi-automatically by first using the “KlustaKwik” algorithm^39^. Unit clustering was then manually refined by cluster cutting performed using “Klusters” (Lynn Hazan, Buzsáki lab, Rutgers, Newark NJ). Only single unit pairs with a geometric mean firing rate of above 1 spike/s 5 were used for the analysis.

### Computation of correlation coefficients

The average of MUA activity over the initial 800 ms of baseline data in a trial was normalized through z-scoring by subtracting the mean of all the average MUA baseline values for that session and then dividing by their standard deviation. The Pearson’s correlation coefficient between channels for these values was then calculated using the trial-by-trial data from all included sessions either pre or post lesion. For calculating the correlation coefficient over a short time window, the baseline was divided into 16 50 ms segments and the average MUA activity calculated for each. To remove the influence of longer time scale rate fluctuation, the correlation across these segments was calculated for each trial and then averaged across all the trials of a session. This value was then averaged across all sessions in the week pre lesion and separately across all sessions in the week post lesion.

To compute spike train correlations methods were as used in Renart *et al.*^5^. On each trial pairs of simultaneously recorded spike trains were first convolved with a Gaussian of standard deviation 25 ms to produce the “Fast Activity”. The Pearson’s correlation coefficient between these convolved spike trains was then calculated for each single trial. The instantaneous mean, calculated by convolving the spike train with a Gaussian of standard deviation 100 ms, was used in the calculation of the Pearson’s correlation coefficient instead of the mean to remove influences of longer time scale fluctuations. Correlation coefficients were then averaged across multiple trials for a single pair to result in the spike train correlation value for a pair. The use of an instantaneous mean and averaging over separate trial segments should remove any correlations resulting from slow firing rate fluctuations on long time scales.

## Additional Information

**How to cite this article**: Shapcott, K. A. *et al.* Correlated activity of cortical neurons survives extensive removal of feedforward sensory input. *Sci. Rep.* **6**, 34886; doi: 10.1038/srep34886 (2016).

## Supplementary Material

Supplementary Information
