# Supplementary material for: Correlated activity of cortical neurons survives extensive removal of feedforward sensory input
Source: Sci Rep. 2016 Oct 10;6:34886. doi: 10.1038/srep34886 (PMC5056506; doi:10.1038/srep34886)
Supplement: Supplementary Information [file srep34886-s1.pdf]

Correlated activity of cortical neurons survives extensive removal of feedforward sensory input

Katharine A. Shapcott[1], Joscha T. Schmiedt[1], Richard C. Saunders[2], Alexander Maier[3], David A. Leopold[2,4], *Michael C. Schmid[1,5]

1. Ernst Strüngmann Institute (ESI) for Neuroscience in cooperation with Max Planck Society, 60528 Frankfurt, Germany

2. Laboratory of Neuropsychology, National Institute of Mental Health, Bethesda, Maryland 20892, USA

3.Vanderbilt University, Department of Psychology, Nashville, Tennessee 37240, USA

4. Neurophysiology Imaging Facility, National Institute of Mental Health, National Institute of Neurological Disorders and Stroke, and National Eye Institute, Bethesda, Maryland 20892, USA

5. Institute of Neuroscience, Newcastle University, Framlington Place, Newcastle upon Tyne, NE2 4HH, UK

* michael.schmid@ncl.ac.uk.

Supplementary Figures

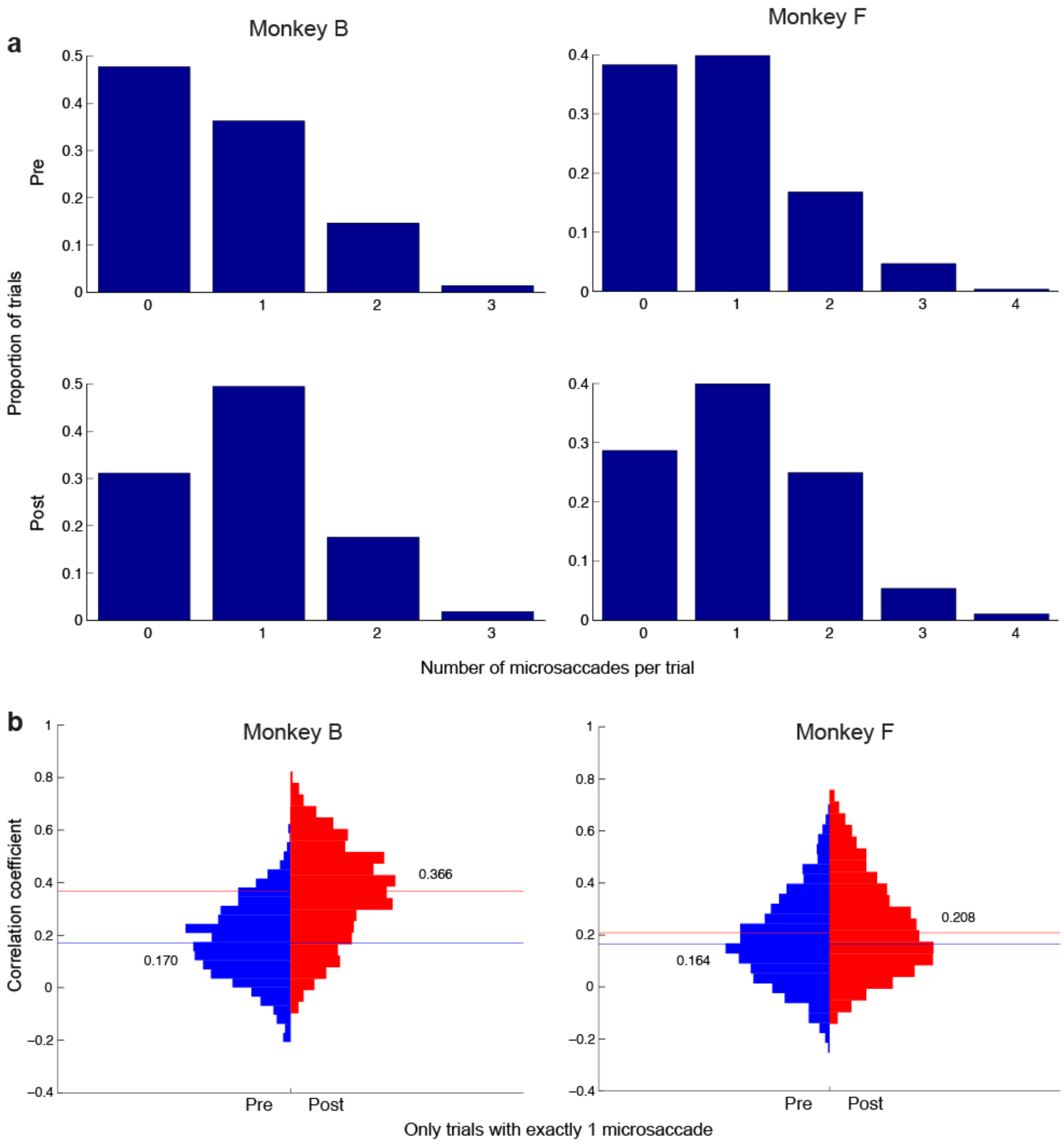

Supplementary Figure S1

Fixational eye movements do not cause correlation increase post lesion. ***a***. Bar plots of the proportion of trials with different numbers of microsaccades pre and post lesion. Only in monkey B is there a significant change from 1 week pre to 1 week post lesion (chi-squared test; monkey B, $p<0.05$ and monkey F, $p=0.444$) ***b***. Recalculated interneuronal correlation coefficients using only trials with one microsaccade (the mode for both monkey B and F). The results are qualitatively similar to those in Figure 2A: in both monkeys there is a significant correlation increase from pre to post lesion (Wilcoxon rank sum; $p<0.001$ and $p<0.001$). Lines and values are the medians for pre and post, respectively.

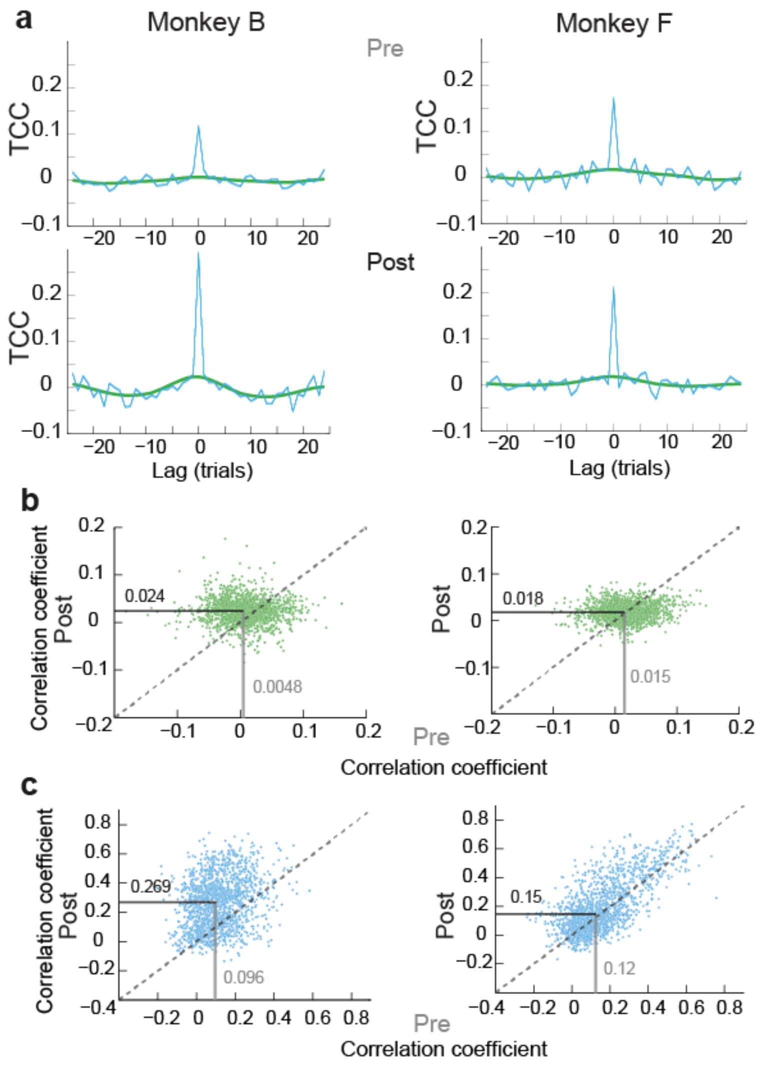

Supplementary Figure S2

Both long term as well as short term components of trial-by-trial interneuronal correlations increase from pre to post lesion. ***a***, Plot of trial cross-covariance (TCC) for pre and post lesion as used by Bair et al. 2001 to examine correlations over time periods greater than one trial[1]. The blue unsmoothed line is the TCC for pre and post lesion, see Supplementary Methods for calculation. The sharp central peak shows that most correlation is due to correlation within a time scale of one trial (~3 seconds average trial length). The smooth green line is a measure of the long term correlation arising over the course of around 4 trials (~12 seconds) see Supplementary Methods for calculation. While this is a small contribution it also increases from pre to post lesion. ***b,*** Long term (~4 trials) correlation contribution significantly increases in both monkeys from pre to post lesion (Wilcoxon rank sum; monkey B, $p<0.001$ and monkey F, $p<0.01$). Lines and values are the median for pre and post. ***c***, Short term (1

trial) correlation contribution also significantly increases from pre to post lesion (Wilcoxon rank sum; $p<0.001$ and $p<0.001$). Short term contribution is estimated by subtracting the long term component from the peak of the TCC at trial 0. Note the larger contribution of the short term component shown by the change in axes scale. Lines and values are the medians for pre and post, respectively.

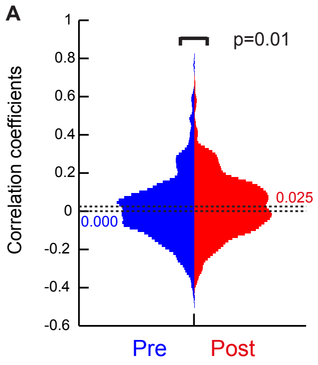

Supplementary Figure S3

Single unit trial-by-trial spike count correlations increase from pre to post lesion. This is significant when tested with Wilcoxon rank sum test ($p=0.011$). Lines and values are the median pre and post correlations for single unit pairs assessed in monkey B.

Supplementary Methods

Computation of spike count correlation

To calculate the spike count correlation, the spikes were summed over the baseline period for each trial. The Pearson's correlation coefficient between these counts per trial was then calculated for simultaneously recorded single units with more than 0.5 spikes per trial.

Microsaccade detection

Microsaccades were detected during the analysis period as in Engbert and Kliegl, 2003[2] using code adapted from the Microsaccade Toolbox[3]. Microsaccades were defined as events with a velocity of over 5 standard deviations from the median on each trial and lasting for a minimum of 3 samples (15 ms for our eye data sampling rate of 200Hz). Detected microsaccades were rejected if they were within 50ms of the previously detected microsaccade as overshoots or if the velocity was greater than

165 visual degrees per second as artifacts. The comparison of microsaccade number between pre and post lesion was assessed using a two-sample chi squared test.

Computation of Trial Cross Covariance (TCC)

The TCC was calculated as in Bair et al. 2001[1]. The cross-correlation of the average MUA spiking activity was calculated for each session and then first averaged across all sessions pre lesion and then all sessions post lesion for each monkey. To calculate the long term component, the TCC at the 0 trial point was replaced with the mean of the -1 and 1 trial lag and then was smoothed with a Gaussian with a 4 trial standard deviation. This value of the smoothed line at trial 0 is the long term component of the correlation. To estimate the short term component of the correlation we subtracted the long term component from the value of the unsmoothed TCC at trial 0.

Supplementary References

1. Bair, W., Zohary, E. & Newsome, W. T. Correlated firing in macaque visual area MT: time scales and relationship to behavior. *J. Neurosci.* **21,** 1676–97 (2001).

2. Engbert, R. & Kliegl, R. Microsaccades uncover the orientation of covert attention. *Vision Res.* **43,** 1035–1045 (2003).

3. Engbert, R., Sinn, P. & Mergenthaler, K., & Trukenbrod, H. (2015). Microsaccade Toolbox. (2015).
